# Berberine Interferes with the Molecular Landscape of Biofilm-Driven Pathogenicity

**DOI:** 10.3390/pathogens15020194

**Published:** 2026-02-10

**Authors:** Anna Duda-Madej, Hanna Bazan, Jakub Łabaz, Szymon Viscardi

**Affiliations:** 1 Department of Microbiology, Faculty of Medicine, Wroclaw Medical University, Chałubińskiego 4, 50-368 Wroclaw, Poland; 2 Faculty of Medicine, Wroclaw Medical University, Ludwika Pasteura 1, 50-367 Wroclaw, Polandjakub.labaz@student.umw.edu.pl (J.Ł.); szymon.viscardi@student.umw.edu.pl (S.V.)

**Keywords:** antibiofilm, antimicrobial resistance, berberine, biofilm, efflux pumps, multidrug-resistant, quorum sensing inhibitors

## Abstract

Biofilm-associated infections pose a significant clinical challenge due to their increased antibiotic tolerance and strong association with multidrug-resistant pathogens. The biofilm protects bacteria against antimicrobial agents and host immune response through a complex matrix, altered cell metabolism, activation of quorum sensing, and overexpression of efflux pumps. Despite the availability of numerous therapeutic strategies, the effectiveness of treatment of these infections remains limited, justifying the search for new pharmaceutics, e.g., compounds of natural origin. Berberine, an isoquinoline alkaloid from the plants of the *Berberidaceae* family, is of growing interest due to its broad spectrum of antimicrobial and antibiofilm activities. This review summarizes the current state of knowledge regarding the molecular mechanisms of action of berberine against the biofilm forming Gram-(+) and Gram-(−) bacteria. Its effect on bacterial cell adhesion, modulation of quorum sensing, inhibition of extracellular matrix synthesis, disruption of biofilm maturation, and the dispersion process are discussed. The role of berberine as an adjuvant to antibiotic therapy was also analyzed, in particular, its ability to restore bacterial sensitivity to different classes of antibiotics. The pharmacokinetic limitations of berberine and the prospects for the use of modern delivery systems are also considered. The collected data indicate that berberine is a promising factor supporting the treatment of biofilm-related infections.

## 1. Introduction

A biofilm is a highly organized community of bacterial cells surrounded by a self-produced polymeric matrix composed of polysaccharides, proteins, and extracellular DNA (EPS). The ability to form this three-dimensional structure is exhibited by both Gram-(+) and Gram-(−) bacteria, and its formation is most often associated with pathogenic potential and the hospital environment. Biofilm development is a complex, multistage process comprising (i) reversible attachment to a surface; (ii) irreversible attachment; (iii) microcolony formation; (iv) development of three-dimensional structures; and (v) maturation followed by detachment and dispersal from the occupied surface. This irreversible phenomenon occurs particularly in the presence of permanent medical devices, including heart valves [1], intravenous catheters [2], endotracheal tubes [3], stents [4,5], intrauterine devices [3,6], pacemakers [7], joint prostheses [6], and urinary catheters [5], and is also observed in up to 75% of chronic wounds [8]. The microenvironment within this architecturally complex microbial community is unique and provides bacteria with multiple advantages, including (i) enhanced biodiversity [9]; (ii) efficient intercellular communication via quorum sensing (QS) [10,11]; (iii) extensive horizontal gene transfer [12,13]; (iv) maintenance of accumulation of virulence-associated extracellular enzymes [14]; (v) recycling of nutrients [15]; and (vi) high protection against environmental stress (e.g., temperature fluctuations, drying, nutrient deprivation, UV radiation, disinfectants, and antibiotics) [16,17,18,19], and the immune system [20]. Therefore, its presence is a key factor in the pathogenesis of chronic and biomaterial-associated infections, especially those caused by MDR (multidrug-resistant) pathogens [21,22]. According to epidemiological data provided by the National Institutes of Health (NIH), as much as 65% of all bacterial infections and approximately 80% of all chronic infections are associated with biofilm formation [23], occurring on both non-living and living surfaces (inert, non-living materials or living tissues) [3].

**Figure 1:**
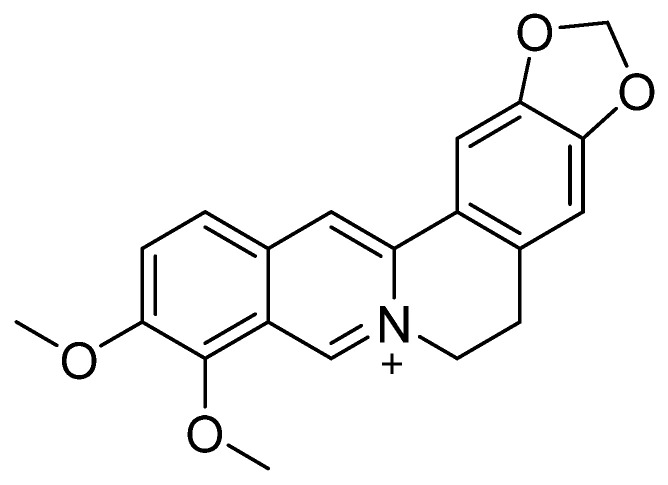
Molecular structure of BBR.

Currently, a broad spectrum of therapeutic strategies targeting biofilms is available, including a wide range of antibiotics, QS inhibitors, and EPS-degrading compounds. However, their clinical effectiveness is limited due to low penetration of the biofilm matrix and phenotypic heterogeneity of biofilm-embedded bacteria [24,25,26]. In recent years, increasing attention has been directed toward natural plant-derived compounds as modulators of the early stages of biofilm formation, offering a promising alternative strategy. One of the most extensively studied compounds in this context is berberine (BBR), an isoquinoline alkaloid and quaternary ammonium cation [27], naturally occurring in plants of the *Berberidaceae* (e.g., *Berberis aristata, Berberis vulgaris*), *Ranunculaceae* (e.g., *Coptis chinensis, Hydrastis canadensis)*, and *Rutaceae* (e.g., *Phellodendron amurense*) families [27,28,29]. The chemical structure of BBR is presented in Figure 1.

Physicochemical features: molecular formula C_20_H_18_NO_4_^+^ (molar mass 336.4 g), yellow powder, poorly soluble in water, high in organic solvents, most often occurring in the form of salts—chlorides or sulfates. The physicochemical properties of BBR influence the low bioavailability of the preparation after oral administration [30].

**Figure 2:**
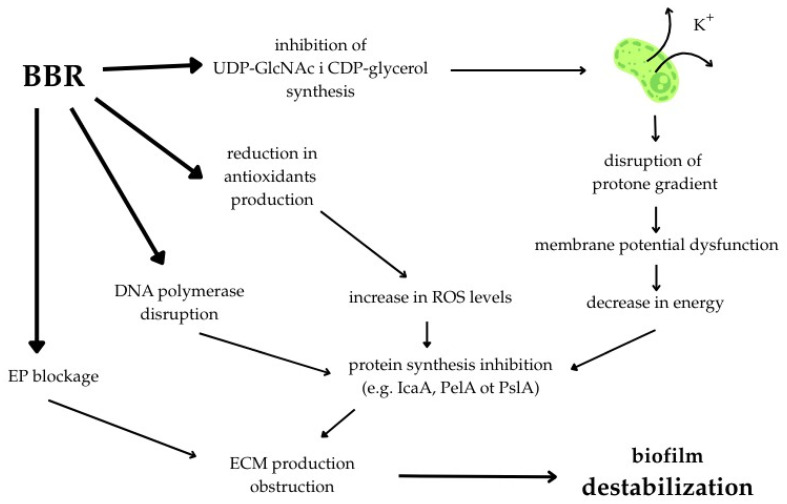
The broad-spectrum anti-biofilm activity of BBR as therapeutic agent. Abbreviations: CDP-glycerol—cytidine-glycerol diphosphate; DNA—deoxyribonucleic acid; ECM—extracellular matrix; EP—efflux pump; K^+^—potassium ion; ROS—reactive oxygen species; UDP-GlcNAc—uridine diphosphate N-acetylglucosamine.

Studies indicate that the alkaloid exhibits broad anti-inflammatory mechanisms [31,32], which have been confirmed in animal in vivo models of inflammatory diseases such as IBD (inflammatory bowel disease), NAFLD (non-alcoholic fatty liver disease), and AD (Alzheimer’s disease) [33,34,35]. Alkaloid is also known for its antioxidant and antineoplastic activity. Exposure of various cancer cell lines to alkaloid in vitro and in vivo reduced tumor proliferation across multiple cancer models (colorectal, gastric, melanoma, glioblastoma, pancreatic, and breast cancer) [36,37,38,39,40,41]. Additionally, the alkaloid demonstrates antibacterial and anti-biofilm activity, including QS silencing, and inhibition of efflux pumps (EP), e.g., NorA and MexAB-OprM, thereby enhancing the efficacy of several antibiotic classes (β-lactams, aminoglycosides, fluoroquinolones) [42]. It supports intestinal barrier function by (i) increasing mucus-producing goblet cells [43,44], (ii) stabilizing tight junctions [45,46], and (iii) modulating the microbiota toward short chain fatty acid (SCFA)-producing species while reducing potentially pathogenic strains [43,47,48]. This broad mechanistic spectrum distinguishes it from other natural antimicrobial compounds. The broad-spectrum anti-biofilm activity of BBR was presented in Figure 2.

The information gathered in this review suggests that, due to well-documented antipathogenic activity against Gram-(+) and Gram-(−) bacteria [36,49,50], future research on BBR should integrate all of its multiple antimicrobial-adjacent activities to more accurately prove its preventive potential against biofilm-associated infections. It remains important to understand the mechanisms by which this alkaloid can be a potential anti-infective pharmaceutical. This review aims to integrate these aspects at the molecular level, including the suppression of QS pathways, disruption of EP activity, and inhibition of bacterial adhesion in the context of biofilm-associated infection prevention. We summarize the current state of knowledge regarding the mechanisms of BBR action at all stages of biofilm formation. In turn, we discuss its antibiofilm potential from the perspective of its use in anti-infective therapy in the era of increasing antibiotic resistance.

## 2. Biofilm Formation and Its Clinical Implications

### 2.1. Biofilm Structure and Development

Bacterial cells can exist in the environment in two forms: free-living planktonic forms and sessile forms with biofilm formation, providing enhanced adaptation to unfavorable environmental conditions [51,52]. Bacterial biofilm is a complex structure composed mainly of water (~90%) and bacterial cells (~10%), embedded in a matrix of organic and inorganic compounds [53]. The dominant organic component is polysaccharides (called EPS exopolysaccharides) such as alginate, cellulose, and polysaccharide intercellular adhesin (PIA), which form a specific network stabilizing the biofilm matrix. Polysaccharides can be positively charged by magnesium or calcium ions or take the form of polyanions or electrically neutral polymers [51,54,55]. Biofilms may also contain significant amounts of organic acids, e.g., uronic acid or pyruvate [56]. It is now known that the production of EPS is a key issue for the formation of sessile bacterial colony structures, resistant to unfavorable physical factors or, for example, drug penetration [57,58].

**Figure 3:**
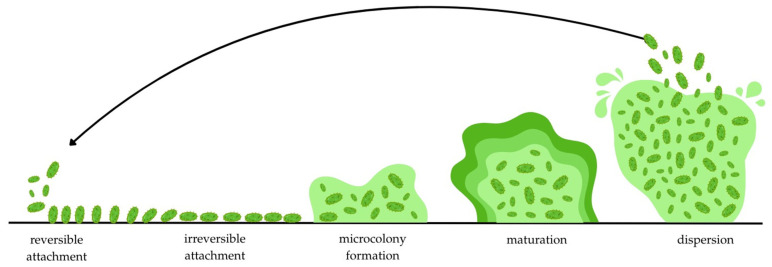
Process of biofilm formation (simplified scheme).

The biofilm formation process is structured and typically involves five steps: reversible attachment, irreversible attachment, microcolony formation, maturation, and dispersal (Figure 3). During the first stage, which depends on temperature, surface type, and pH [59], cells adhere reversibly to living or non-living surfaces using pili, fimbriae, and physicochemical interactions [60,61]. Then, as a result of prolonged adhesion, c-di-GMP increases in the presence of hydrogen, ionic, and dipole–dipole interactions, triggering the synthesis of structures necessary to stabilize intercellular connections [62,63,64]. Intensive cell division, which follows the previous stage, leads to the formation of microcolonies, the basic building blocks of biofilm, and the activation of complex intercellular communication mechanisms based on QS [65,66,67]. The biofilm then matures and reaches a size of ~100 μm. At this stage, there is overproduction of the extracellular matrix (ECM), cell aggregation, and preparation for the dispersion phase [68,69,70,71], in which cells, in response to environmental factors (passive physical factors; active internal mechanisms, e.g., nutrients, O_2_, pH change) return to their planktonic form [72,73].

The key virulence factors/resistance mechanisms associated with subsequent stages of biofilm formation are summarized in Table 1.

### 2.2. Role of Biofilm in Antimicrobial Resistance

Biofilm-forming bacteria exhibit markedly increased resistance to antimicrobials, being 10–10,000 times more resistant to drugs than planktonic forms [16]. Furthermore, structural differences between Gram-(+) and Gram-(−) bacteria, including surface glycopolymers, polysaccharides, and LPS (lipopolysaccharide), play a key role in biofilm formation and resistance [74]. Antibiotic/chemotherapeutic penetration into biofilms is significantly reduced, as demonstrated for β-lactam antibiotics and aminoglycosides (tobramycin, gentamicin) in *P. aeruginosa* due to binding to alginate [75,76,77,78]. The maturation stage creates additional barriers to the penetration of antimicrobial agents, as confirmed by studies by Tseng et al., which showed that the migration of tobramycin in the biofilm of *P. aeruginosa* biofilm was effectively prevented by electrostatic interactions between negatively charged EPS molecules and the cationic antibiotic molecule [79]. The antibiotic perfectly eliminated bacterial cells, but was unable to effectively combat structures located deeper, unlike ciprofloxacin, which has a neutral structure [79].

Additional resistance mechanisms involving acidification of the biofilm environment by extracellular DNA (eDNA), activation of the PhoPQ and PmrAB regulatory systems, and reduced tobramycin penetration have been observed in studies [80,81]. In addition, EPS-related components such as β-lactamases present in the biofilm matrix may further reduce the effectiveness of antibiotics, as demonstrated by in vitro time-killing studies conducted on a *P. aeruginosa* strain overexpressing β-lactamases [82]. These data emphasize that the composition of EPS is not passive, but actively supports the development of antibiotic therapy through chemical and enzymatic changes. Therefore, changes in its composition, e.g., disruption of PEL polysaccharide synthesis, reduce the bactericidal efficacy of antibiotics by up to 4–8 times [83]. In addition, many studies have shown that the phenotypic heterogeneity of biofilm, including the presence of dormant spore cells, promotes reduced tolerance to antibiotics that act on dividing cells [84,85,86,87].

The EPs play a key role in biofilm function. They have been shown to enable the transport of QS molecules, siderophores, and antibiotics [6,88,89,90,91], constituting an early, low-level mechanism leading to the development of high-level resistance [92]. Overexpression of RND family pumps is characteristic of ESKAPE pathogens (including *P. aeruginosa*, *Acinetobacter baumannii*, *Enterobacter* spp., *Klebsiella pneumoniae*) and promotes the development of MDR [93]. This confirms that EPs are a universal and early defense mechanism of biofilms, independent of species. This is a serious clinical problem due to the wide spectrum of substrates of these pumps that determine MDR resistance. This is undoubtedly facilitated by the biofilm environment, which promotes horizontal transfer of resistance genes, e.g., those encoding β-lactamases or the *vanA* gene, making it a more stable reservoir of mobile genetic elements (MGEs) than planktonic forms, e.g., in *A. baumannii*, *Enterococcus faecalis*, and *S. aureus* [94,95,96,97,98]. These observations indicate that biofilms act as a “genetic reservoir”, promoting stable and efficient exchange of resistance genes.

The most important mechanism involved in the regulation of all stages of bacterial biofilm development is the QS system. It is assumed that it is activated at the microcolony formation stage, where, under the influence of various autoinducers (e.g., AHL (N-acetyl-homoserinolactones), PQS (*Pseudomonas* quinolone signal), and AI-2 (autoinducer 2)), it promotes the intensification of EPS synthesis, phenotypic differentiation of cells, and stabilization of the three-dimensional architecture of the biofilm structure [99,100,101]. In subsequent phases, it plays a key role in inducing changes in the EPS structure, facilitating the spread of bacteria [102]. QS also likely induces the degeneration of some biofilm-forming cells, which may facilitate the active dispersion of the remaining cells [103]. Furthermore, QS not only regulates biofilm architecture, but also enables pathogens to effectively evade the host immune response by modulating virulence factor expression, polarizing macrophages from Mφ1 to anti-inflammatory Mφ2, and inhibiting inflammatory pathways, e.g., PAMP/TLR, NF-κB [104,105,106,107,108]. The effect of reduced expression of pro-inflammatory cytokines (e.g., INF-γ, TNF-α, IL-6) [107] contributes to the proliferation of infection and the development of chronic inflammation during the dispersion phase. Limited space within the biofilm structure and poor nutrient availability trigger active biofilm degradation. These mechanisms include the secretion of matrix-degrading enzymes, reduced EPS synthesis, and disruption of non-covalent interactions within the matrix, leading to the release of planktonic cells [66] and initiating another cycle of biofilm formation. The summary of resistance associated with biofilm and its linking with current types of infections is presented in Table 2.

## 3. Antimicrobial Activity of Berberine

### 3.1. Effect on the Cell Wall and Membrane

Metabolomic and multiomic studies have shown that BBR and its derivatives disrupt the integrity of the cell wall and bacterial membrane in both Gram-(+) and Gram-(−) bacteria. Wu et al. demonstrated that exposure of *S. aureus* to BBR hydrochloride led to a decrease in the synthesis of D-alanyl-D-alanine and farnesyl diphosphate synthesis and accumulation of peptidoglycan precursors and teichoic acids, e.g., UDP-GlcNAc, UDP-ManNAc, CDP-ribitol, and CDP-glycerol, strongly inhibiting cell wall biosynthesis [49]. In turn, proteomic analyses conducted by Du et al. confirmed reduced synthesis of Mur enzymatic proteins (MurA2, MurD, MurG) involved in peptidoglycan synthesis [111]. Zhang et al. indicated significant disturbances in the cell membrane cohesion of methicillin-resistant *S. aureus* (MRSA) exposed to BBR concentrations below the MIC (minimal inhibitory concentration) in vitro. At a concentration equal to the MIC (128 μg/mL), a ~44% increase in transmembrane K+ ion leakage and changes in fatty acid composition were observed, leading to the accumulation of cell membrane damage in this strain [112]. Research by Zhao et al. provided additional information on the disruption of the transmembrane proton gradient by this alkaloid, which supports this process [113]. Mechanisms destabilizing bacterial envelopes have also been reported in Gram-(−) bacteria. Karaosmanoglu et al. demonstrated that exposure of *Escherichia coli* to BBR led to global disruption of key metabolic pathways and disruption of gene expression encoding membrane structures, e.g., OmpC and NmpC [114]. BBR derivatives also significantly disrupted cell membrane integrity in both Gram-(−) [115] and Gram-(+) [116] bacteria.

These mechanisms induced by BBR and its derivatives reduce peptidoglycan and teichoic acid synthesis, leading to a significant reduction in cell wall integrity, which directly reduces the ability of bacteria to adhere to surfaces—a key step in biofilm initiation. At the same time, increased cell membrane permeability and the ability of BBR to disrupt the proton gradient impair the energy processes necessary for ECM synthesis and biofilm maturation. Global metabolic and proteomic changes, including decreased expression of Mur enzymes and membrane proteins, further destabilize the structural organization of the biofilm and increase the sensitivity of biofilm cells to environmental factors and antibiotics.

### 3.2. Inhibition of Nucleic Acids and Proteins Synthesis

The effect of alkaloids on the reduction of organic compound synthesis, such as proteins and nucleic acids, has been widely documented by various researchers [49,114,115]. In a study by Du et al., the proteome of *S. pyogenes* was evaluated after exposure to BBR, revealing disturbances in the synthesis of proteins responsible for carbohydrate metabolism (including the pentose phosphate pathway, fructose and mannose metabolism), peptidoglycan synthesis, amino sugar and nucleotide metabolism, pyruvate metabolism, and fatty acid metabolism, as well as DNA replication, transcription, and ribosome biogenesis [111]. In *E. coli* K12, this alkaloid caused a decrease in the expression of genes responsible for carbohydrate and amino acid metabolism, as well as the synthesis and metabolism of energy carriers and oxidoreductase enzymes [114]. In addition, BBR induced a more than 10-fold decrease in the expression of the *cadA* and *cadB* genes encoding essential *E. coli* virulence proteins. The production of the FtsZ protein, responsible for bacterial cell division, was also impaired [114]. In turn, BBR derivatives showed affinity for DNA polymerase III and disrupted the structure of the DNA double helix, inhibiting replication [115]. Protein synthesis disorders can also be observed in the inhibition of amino acid synthesis by BBR. A study by Wu et al. indicated that the alkaloid can lead to the inhibition of the synthesis of aromatic amino acids L-phenylalanine, L-tyrosine, and L-tryptophan in S. aureus through the accumulation of chorismate, their bacterial precursor [49].

Therefore, BBR induces multilevel metabolic and molecular disturbances that directly disrupt key stages of biofilm formation. Inhibition of protein and nucleic acid synthesis leads to a reduction in the production of adhesive proteins, enzymes responsible for ECM synthesis, and biofilm gene expression regulators. Simultaneous disturbances in carbohydrate and energy carrier metabolism limit the availability of precursors and energy necessary for the synthesis of biofilm polysaccharides. In addition, disturbances in normal protein folding and cell division destabilize the spatial organization of the bacterial population, leading to inhibition of biofilm initiation, slowing of its maturation, and reduced stability of the mature biofilm structure.

### 3.3. Efflux Pump Inhibition

Numerous studies have shown that BBR and its derivatives can be used as inhibitors of the main EP families: MFS, RND, and ABC, in both Gram-(+), Gram-(−) bacteria, and *Mycobacterium* spp. [42]. This alkaloid restored antibiotic sensitivity by directly blocking the MdfA pump in *E. coli* [117] and NorA, NorB, and MepA in *S. aureus* [118]. The anti-efflux activity of BBR has been well documented in pathogens expressing pumps from the RND family (MexXY-OprM, AdeABC, AcrAB-TolC) in both the Enterobacteriaceae family and non-fermenting bacilli, e.g., *P. aeruginosa* and *A. baumannii* [117,119,120,121,122], which is crucial in overcoming MDR resistance, common in these bacteria. In addition, this alkaloid has also shown a wide range of inhibitory abilities for EPs present in *Mycobacterium avium-intracellulare* complex (MAC) isolates [123]. In the study by Schildkraut et al., it was mentioned that BBR reduced efflux activity in isolates carrying ABC, MFS, and RND (MmpL) EPs, and that the substance in combination with clarithromycin induced a synergistic interaction [123].

Therefore, BBR and its derivatives, by directly blocking the activity of EPs (MFS, RND, ABC) and reducing the expression of genes encoding their key subunits, increase the intracellular accumulation of antibiotics and toxic compounds. In the context of biofilm, this leads to disruption of QS molecule transport, interference with stress tolerance mechanisms, and weakening of the protective role of biofilm as a diffusion barrier, as well as disruption of population coordination and biofilm maturation. It is particularly important that BBR, like other EP inhibitors, exhibits protective effects at concentrations below the MIC. In addition, restoring the sensitivity of biofilm cells to antibiotics increases their susceptibility to elimination, which destabilizes the structure of mature biofilm and limits its ability to survive in the clinical environment.

### 3.4. Energy Metabolism Disorders and Oxidative Stress in the Bacterial Cell

Previous studies have shown that BBR causes significant redox imbalances in bacterial cells. Research by Wu et al. showed that this effect in *S. aureus* was associated with a reduction in the synthesis of antioxidant compounds, including 2,5-pyridinedicarboxylic acid, 2,6-pyridinedicarboxylic acid, and γ-tocopherol [49]. The accumulation of oxidized glycerol phospholipid clearly indicated the failure of oxidative stress compensation mechanisms, and these reports are reflected in the study by Du et al., which showed that exposure of *S. pyogenes* to BBR induced the accumulation of ROS (reactive oxygen species) resulting from the disruption of key carbohydrate metabolism pathways (e.g., fructose and mannose metabolism, glycolysis, gluconeogenesis). Importantly, the presence of antioxidants such as N-acetyl-L-cysteine significantly reduced the antibacterial efficacy of this alkaloid [111]. This effect is enhanced by the disruption of carbohydrate metabolism, which, in combination with the above-mentioned mechanisms, exacerbates oxidative damage to DNA and membrane lipids, leading to bacterial cell death [112].

Therefore, BBR disrupts bacterial redox homeostasis by significantly reducing the synthesis of antioxidant compounds. This leads to uncontrolled accumulation of ROS, resulting in impaired membrane integrity, adhesion, and biofilm architecture. In turn, disturbances in energy metabolism limit the adaptive capacity of biofilm cells. As a consequence of these actions, the biofilm loses its protective function and ability to mature, resulting in increased sensitivity to antibiotics and environmental factors.

The presented data on the antimicrobial and antibiofilm activity of BBR are primarily derived from in vitro studies, which may not fully reflect the complexity of in vivo conditions, including host–pathogen interactions and microbial community dynamics. In addition, numerous mechanistic effects were observed at concentrations that were close to or above the MIC, which raises concerns about their clinical significance given the limited bioavailability of BBR. Another limitation is the heterogeneity of experimental models and bacterial strains used across studies, which may affect the comparability and generalizability of the results. Additionally, although multiomics approaches provide valuable insights into global metabolic and regulatory changes, these findings are largely correlative and do not always establish direct causal relationships between specific molecular targets and antibiofilm activity. It is necessary to conduct additional in vivo and pharmacokinetic studies to assess BBR’s therapeutic applications due to its potential cytotoxicity towards eukaryotic cells and its impact on beneficial microbiota.

## 4. Influence of Berberine on Biofilm Formation

### Berberine as a Biofilm Formation Inhibitor

Multiple experiments have been carried out in order to implore BBR’s effectiveness against bacteria, fungi, and their biofilm formation. They not only confirmed this alkaloid’s efficacy in eliminating microorganisms’ biofilms and ability to synergize with antibiotics, but also discovered some of the mechanisms behind BBR’s success. It appears that it can influence each of the biofilm formation stages by many different mechanisms.

At the outset, it was observed that BBR influences the AI-2 signaling molecule path, reducing its synthesis [124]. This protein plays a crucial role in the adhesion of bacteria, as it regulates the QS system [125]. Its absence disrupts the whole attachment stage, thus preventing microorganisms from creating biofilm. BBR’s involvement in bacterial adhesion has also been noted in another study regarding those pathogens, although the exact mechanism has not been stated there [126].

There is also some evidence of BBR’s influence on the growth and maturation stage as it interacts with multiple *S. aureus* genes, reducing their synthesis, thus halting biofilm development [118,127]. Identified fragments of genomes are listed and described below in Table 3.

It has also been observed that inhibition of those genes results in impaired formation of amyloid fibrils in the biofilm [118,134]. They are highly stable protein aggregates built with phenol-soluble modulins (PSMs). Those fibrils provide a scaffold for biofilm formation, thus contributing to bacterial community cohesion, physical stability of biofilm, and its environmental resistance [135]. BBR was found to bind with the phenyl ring in PSMα2, thereby influencing its aggregation ability [136]. As those compounds interact, biofilm loses its three-layered structure, and therefore, its functionality [137]. It is also worth mentioning that BBR’s influence on the *agr* system negatively impacts the last step of biofilm formation—its dispersal [138].

BBR appears to interact with *P. aeruginosa* biofilm as well. An in silico study showed that it can interact with two proteins important for biofilm formation—LasR and RhlR [126]. They are central QS regulators in *P. aeruginosa*. LasR typically initiates the QS cascade, enabling RhlR expression. They both coordinate behaviors such as virulence factor production or biofilm formation [139]. Similar observations—biofilm reduction—were made when BBR was used against this bacteria in combination with antibiotics such as azithromycin—both in vitro and in vivo in mouse models [140,141]. This also resulted in lowered expression of the regulatory genes *algG*, *algD*, and *algR*, which play a role in alginate synthesis: its initiation, product modification, and process regulation [140]. As *P. aeruginosa* biofilm is rich in alginates, reduction in their quantity may be one of the potential strategies against this bacteria [142]. There is also evidence that BBR is interacting with *pslA* and *pelA* genes [19]. In *P. aeruginosa*, *pslA* and *pelA* encode for the core exopolysaccharide synthesis pathways (Psl and Pel) that build and stabilize the biofilm matrix. Psl is the main staging, which drives biofilm adhesion and architecture, while Pel provides additional structural support, biomass, and resilience—especially under stress or when Psl is impaired. [143].

What is interesting is that BBR has also been active against adhesion of fungi from *Candida* species, when paired with an antibiotic. In a synergy with fluconazole, this alkaloid reduces synthesis of adhesion-related genes such as ALS3 or HWP1 [144,145]. Yeasts devoid of the correct *ALS3* or *HWP1* gene cannot form a proper biofilm and their cell-to-cell adhesion is decreased [146,147]. It also appears that, with an antibiotic, BBR may be able to inhibit growth of planktonic cells of this fungi and, importantly, cells in biofilms, thus reducing their virulence [148]. Its bactericidal and anti-biofilm properties seem to be enhanced as biofilm matures [149]. This may positively influence healing processes.

It is important to note, though, a problem that currently hampers BBR’s introduction as a new anti-biofilm chemotherapeutic agent. It has been noticed that to work properly, BBR must achieve a desired concentration in an instant, as otherwise it may trigger a mechanism similar to antibiotic resistance or even improve biofilm formation [150].

## 5. Berberine as an Adjunct to Conventional Antibiotic Therapy

The concept of combining antibiotics with adjuvants—compounds that exhibit little or no intrinsic antimicrobial activity but are capable of restoring or enhancing antibiotic efficacy against resistant strains—has gained increasing attention [151]. Based on the mechanisms underlying their synergistic interactions with antibiotics, adjuvants can be broadly classified into three major groups, the characteristics of which are summarized in Table 4. The role of antibiotic adjuvants is of particular importance, as their use may lead to (i) a reduction in selective pressure exerted on MDR bacterial populations, thereby slowing the emergence and dissemination of antimicrobial resistance [102,152], as well as (ii) a decrease in drug-associated adverse effects, including mitigation of the detrimental impact of antibiotics on the gut microbiota [153]. Antibiotic adjuvants encompass a wide range of chemical entities, including synthetic compounds (e.g., peptidomimetics and nanoparticles) as well as naturally derived molecules, such as alkaloids, phenolic compounds, stilbenes, and terpenes [154]. BBR, which belongs to this latter group, exhibits particularly promising antimicrobial activity (see Section 4). However, due to limitations, including pharmacokinetic ones (more on this in Section 7), its monotherapy is often insufficient. Therefore, there is a fundamental question whether a strategy of combining antibiotics and BBR can effectively overcome the problem of growing antibiotic resistance, particularly among bacteria capable of producing biofilms.

### 5.1. Berberine as a Multi-Pronged Adjuvant—Synergistic Combinations with Antibiotics Against Biofilm-Forming Pathogens

#### 5.1.1. In Silico Studies—Mechanistic and Computational Evidence

Before the experimental validation in biological systems, several studies have used in silico approaches to investigate the molecular basis of BBR as an antibiotic adjuvant and biofilm inhibitor. These computational analyses provide early mechanistic insights into the interactions between BBR and key bacterial targets involved in antibiotic resistance and biofilm development, supporting hypotheses subsequently tested in in vitro and in vivo models.

Aswathanarayan et al. conducted molecular docking simulations of BBR with the QS receptors LasR and RhIR in *P. aeruginosa*. Molecular dynamics analysis of these interactions indicated that BBR may interfere with biofilm formation as an indirect antibiotic adjuvant, targeting regulatory pathways rather than exerting direct bactericidal effects [126].

Further in silico studies confirmed and extended these observations, demonstrating stable interactions between BBR and additional QS regulators, including additionally PqsR. Yakobi et al.’s studies supported the idea that BBR’s disruption of bacterial communication networks may contribute to increased antibiotic susceptibility in biofilm-forming pathogens by binding to proteins within the biofilm, providing a structural rationale for its experimentally observed antibiofilm activity [156].

Importantly, the antibiofilm potential of BBR suggested by in silico studies is not limited to interference with QS in Gram-(−) bacteria. Computational analyses also provided mechanistic insights into the effect of BBR on the structural integrity of biofilms formed by Gram-(+) pathogens. In this regard, the study by Chu et al. is particularly noteworthy. They investigated in silico the interactions between BBR and phenol-soluble modulins (PSMs), key amphipathic peptides contributing to the stability and architecture of methicillin-resistant *S. aureus* (MRSA) biofilms. Their simulations demonstrated that BBR can bind to PSMs and modify their conformational dynamics, potentially destabilizing the biofilm structure. This provides a structural explanation for the experimentally observed increased antibiotic penetration and activity against MRSA biofilms in the presence of BBR [136].

Although in silico studies do not reflect the complexity of biological systems, they play an important role in identifying potential molecular targets and guiding experimental studies. In the context of BBR, computational evidence supports its classification as a multi-pronged antibiotic adjuvant through multiple complementary mechanisms, including disruption of regulatory pathways controlling biofilm formation, as well as direct destabilization of biofilm structural components.

#### 5.1.2. In Vitro Research

In silico studies paved the way for the next stages of research, including in vitro studies, which made synergistic combinations of BBR with antibiotics one of the most well-documented examples of using a natural adjuvant to increase the effectiveness of therapy against resistant bacteria, especially against strains capable of forming biofilms. Numerous studies have demonstrated that BBR can act simultaneously as a direct adjuvant, an indirect adjuvant, and—though less frequently described—as a host response modulator, making it a versatile tool supporting conventional antibiotic therapy [50]. It is important to emphasize that most studies of BBR as an antibiotic adjuvant in biofilm-associated infections are based on in vitro models. These studies provide important mechanistic insights but do not fully reflect the complexity of in vivo or clinical infections.

At the level of direct action, BBR enhances the activity of drugs by inhibiting bacterial resistance mechanisms such as EP. Overexpression of the NorA, MepA, NorB, and SdrM pumps is one of the most important factors reducing the efficacy of fluoroquinolones in MRSA infections. Seo et al. demonstrated in their study that BBR, the active substance of the *Corydalis Tuber* extract, can bind to all four EP proteins and abolish the MRSA resistance mechanism [118]. Similar relationships were also observed by Su et al., whose studies showed that the combination of BBR with imipenem inhibited the MexXY-OprM EP in carbapenem-resistant *P. aeruginosa* [157].

BBR also acts as an indirect adjuvant, influencing the biofilm structure and physicochemical properties of the bacterial cell. Research by Zhou et al. showed that BBR can enhance the action of many classes of antibiotics (including β-lactams, quinolones, aminoglycosides, tetracyclines, macrolides, lincosamides, and fusidic acid) in combating Gram-(−) biofilm-forming pathogens, such as *E. coli*, *P. aeruginosa*, *K. pneumoniae*, and *Salmonella* spp. The authors emphasize that one of the key mechanisms of this synergy is limiting the adhesion, microcolony formation, and maturation of biofilm at an early stage [50]. In turn, Zhao et al. demonstrated that combining BBR with azithromycin against *P. aeruginosa* PAO1 reduced the production of alginate, an important component of the biofilm of this bacterium [140].

Accumulating evidence also suggests that BBR may act as a host-modulating adjuvant, further expanding its therapeutic potential. It has been shown to inhibit the production of proinflammatory cytokines (e.g., TNF-α, IL-1β, IL-6) and promote the expression of anti-inflammatory mediators (e.g., IL-10), reducing inflammation and promoting infection control [158]. In mouse models by Xiao et al., BBR increased host immunity, including by activating the p38MAPK pathway, which resulted in a reduction in the number of pathogens in tissues and improved survival [159]. Thanks to its host-modulating effect, BBR can support antibiotic therapy not by directly inhibiting bacteria but by strengthening the immune response, which promotes the elimination of pathogens and limits the development of resistance.

Enhanced antimicrobial efficacy against resistant and biofilm-forming bacteria can be achieved through the use of synergistic combinations of BBR and antibiotics, as documented. BBR can act as both a direct adjuvant and an indirect adjuvant by inhibiting resistance mechanisms like efflux pumps, and as a host response modulator less frequently. The restoration of antibiotic susceptibility in pathogens like MRSA and carbapenem-resistant *P. aeruginosa* by BBR is a result of its ability to inhibit early biofilm formation and reduce biofilm matrix components. In vitro models are the primary source of most findings, which highlight the need for further in vivo and clinical validation.

A summary of examples of synergistic BBR–antibiotic combinations is provided in Table 5.

#### 5.1.3. Preclinical (In Vivo) and Clinical Studies

Although considerable evidence supports the synergistic effects of BBR with conventional antibiotics in vitro, data from preclinical infection models and clinical trials remain limited.

Among the notable preclinical studies, Li et al. demonstrated that BBR significantly enhanced the activity of azithromycin against *P. aeruginosa* isolated from patients with cystic fibrosis of the lung, both in in vitro biofilm assays and in a mouse model of lung infection. In this study, combination therapy reduced bacterial burden, attenuated virulence-related traits, and improved survival outcomes compared to antibiotic monotherapy, providing direct evidence that BBR can act as an antibiotic adjuvant in biologically relevant settings [141]. Further support for this hypothesis can be found in the preclinical studies of Li et al., involving models of MDR-*A. baumannii* infection. In a murine model of neutropenic thigh infection, BBR hydrochloride combined with sulbactam significantly increased the antibiotic’s antibacterial efficacy against the MDR pathogen compared to sulbactam alone [160]. Both animal studies confirm that BBR’s adjuvant effect is not limited to a single pathogen or antibiotic class but can enhance the activity of various conventional drugs in vivo, including in models of biofilm-associated infection.

Clinical evidence is still scarce and currently limited to small pilot studies. A translational study by Mangiaterra et al. demonstrated that BBR significantly reduced the number of antibiotic-tolerant cells in *P. aeruginosa* biofilms from patients with cystic fibrosis and increased the bactericidal activity of tobramycin. Despite the promising study results, these results were obtained using ex vivo biofilm models and clinical isolates from only 20 patients [161]. Similarly, satisfactory results regarding the synergistic effect of BBR in combination with antibiotics (clindamycin and rifampicin) were reported for methicillin-resistant and biofilm-forming clinical isolates of *S. aureus* from infected patients’ bloodstreams, but these observations were limited to laboratory analyses. However, due to the limited sample size (15 MRSA isolates) and the lack of a randomized, controlled trial, these results should be considered preliminary [162].

Overall, although preclinical in vivo studies and early clinical observations provide promising evidence for BBR as a potentiator of antibiotic action in combating biofilm, reliable clinical trials are still lacking. Further translational studies are needed to determine its therapeutic value, optimal dosing strategies, and safety profile in patients with biofilm-associated infections. While there is strong in vitro evidence that BBR and conventional antibiotics synergize, evidence from in vivo infection models and clinical studies is scarce. BBR–antibiotic combinations have been shown to improve antibacterial efficacy and outcomes in preclinical animal studies, but these findings are restricted to certain pathogens and experimental models. The majority of clinical evidence today is limited to small pilot and ex vivo studies with limited sample sizes and non-randomized, controlled trial designs. Consequently, while the available data suggest that BBR may act as a promising antibiotic adjuvant against biofilm-associated infections, its clinical efficacy, optimal dosing, and safety profile remain insufficiently established and require further well-designed translational and clinical studies.

### 5.2. Indications for Combination Therapies with Berberine

Combination therapies involving BBR may be a particularly beneficial strategy in several clinically relevant situations. Importantly, this approach appears promising in the treatment of infections caused by MDR pathogens, where BBR has been shown to enhance antimicrobial efficacy and interfere with resistance mechanisms [50].

BBR-based combination treatment regimens may also be beneficial in the management of chronic wound infections, including pressure ulcers, postoperative wounds, and diabetic foot ulcers. In these conditions, biofilm formation is a major barrier to effective healing by impairing tissue regeneration and promoting recurrent infections. BBR’s ability to disrupt biofilm architecture and sensitize bacteria to antimicrobial agents supports its potential role as an adjunct in chronic wounds management [163,164].

Furthermore, respiratory tract infections caused by *Pseudomonas* spp., particularly in patients with cystic fibrosis, represent another area where BBR may provide therapeutic benefits. *P. aeruginosa* is known for its strong biofilm-forming capacity and natural resistance to antibiotics, and BBR has been reported to impair biofilm development and virulence-associated pathways in this pathogen [161].

Infection associated with medical devices, including orthopaedic implants, urinary and vascular catheters, and prosthetic materials, also constitute a significant clinical challenge due to the rapid establishment of biofilm on abiotic surfaces. In such situations, BBR can be a valuable supplement, limiting the initial adhesion of bacteria, biofilm maturation, and its persistence on biomaterials, thereby improving the long-term effectiveness of implanted devices [165].

Finally, BBR has great potential in prophylactic applications, especially as a component of antimicrobial coatings for biomaterials. The addition of BBR into implant surfaces, hydrogels, wound dressings, or composite systems combining BBR with conventional antibiotics can effectively prevent early-stage bacterial colonization and biofilm formation, thereby reducing the risk of infection from the outset [166].

**Figure 4:**
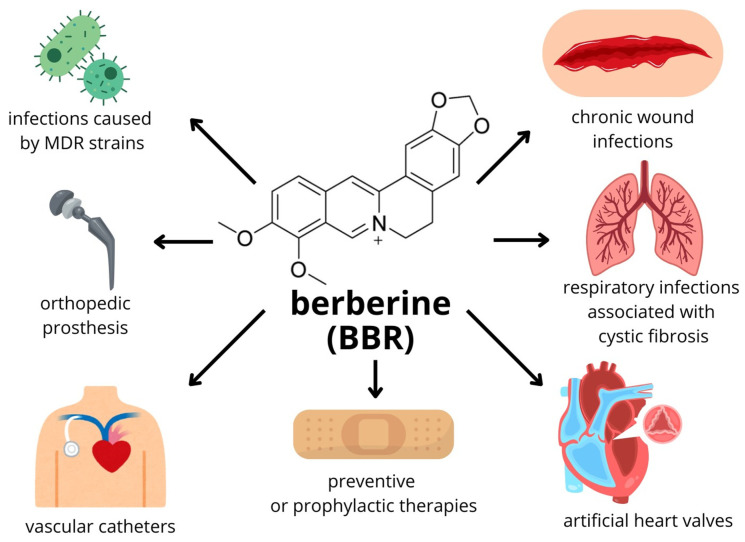
Prospective clinical applications and hypothetical targets for BBR-based combination therapies.

A summary of the indications for combination therapies with BBR is provided in Figure 4.

## 6. Challenges and Recommendations

Despite its well-documented antibacterial and biofilm-inhibiting properties, the practical use of BBR (or rather, its clinically used form—orally administered hydrochloride salt) is limited primarily by the low bioavailability of BBR, and consequently, difficulties in effective delivery to the site of infection. BBR has poor water solubility and a low intestinal absorption rate of less than 5%. This is partially due to the presence of a strongly hydrophilic quaternary ammonium group in its structure, which reduces transmembrane transport of BBR [50,167]. Although BBR is rapidly distributed in various tissues (liver, kidneys, muscle, adipose tissue, lungs, heart, and brain), the low bioavailability following oral administration limits the amount of the compound reaching the circulation and, subsequently, the tissues already at the stage of intestinal absorption. Additionally, extensive first-pass metabolism (CYP2D6, CYP1A2, CYP3A4, CYP2E1, and CYP2C19), active efflux through P-glycoprotein transporters, and efficient excretion of BBR in bile, feces, and urine make achieving an effective dose at the site of infection (e.g., implant-associated biofilm) a significant challenge [30,168]. Another barrier is limited biofilm penetration. Even if BBR reaches the site of infection, its ability to penetrate the biofilm matrix may be narrowed for a number of reasons. These include negatively charged EPS in the biofilm structure [169], variability in the biofilm microenvironment, including pH gradients, affecting the physicochemical properties of EPS and particle diffusion [170], and the above-mentioned activity of EPs in surface layers. Therefore, BBR often proves more effective in preventing biofilm formation than in eradicating already mature biofilms [126,171].

To overcome these barriers, studies have explored a number of advanced delivery systems. Nanocarriers, such as the PEG–lipid–PLGA NPs/BBR-SPC nanoparticles used in the study by Yu et al. 2017, improve the solubility and cell membrane penetration of BBR as well as its stability after oral administration and controlled release, achieving higher tissue concentrations in rat models [172]. On the other hand, metal-based nanoparticles are available. For example, Wang et al. 2025 innovatively combined BBR with silver nanoparticles (AgNPs) and carboxylated chitosan, which significantly enhanced its antimicrobial and antibiofilm activity against MRSA in the treatment of skin infections and positively impacted the wound-healing process [173]. A similar improvement in BBR activity against MRSA was noted by Sadeghi et al. 2024 after combining it with gold nanoparticles (AuNPs) in in vitro studies [174]. Al-Awady et al. 2017 demonstrated that BBR in the form of a nanogel with cationic surface charge increases its antibacterial activity against *E. coli* and *Chlamydomonas reinhardtii* due to enhanced interaction with the cell membrane [175]. There are also studies on local BBR delivery systems in the form of dressings for the treatment of chronic diabetic wounds: a polysaccharide hydrogel with BBR, combining anti-inflammatory, antioxidant, antibacterial, and tissue regeneration-stimulating effects [164], as well as a BBR nanofibrous dressing, which in in vitro and in vivo studies accelerated wound healing and increased angiogenesis and collagen synthesis [176]. A notable way to increase the penetration of BBR into cells, and thus model the immune response profile or combat intracellular pathogens, is to combine an alkaloid with a ligand that facilitates internalization. Feng et al. indicated the benefits of conjugating BBR with yeast antigens, which facilitated the uptake of the compound from the environment by macrophages [31]. Andima et al. demonstrated the effectiveness of combining BBR with lactoferrin against *S. aureus* strain Newman and *Mycobacterium abscessus* [177].

Despite promising preliminary results from preclinical studies, translating them into clinical practice poses a number of challenges. There is a lack of standardized doses—the studies cited above test a wide range of concentrations, making it difficult to determine optimal therapeutic levels. Furthermore, most of these studies were conducted in vitro and few involve animal infection models, while clinical trials are virtually nonexistent. There are also concerns about potential interactions with medications (primarily metabolized by CYP2D6) and the induction of hypoglycemia with simultaneous use of BBR and glucose-lowering medications [178], which requires caution in patients taking multiple medications due to chronic conditions.

It is also important to remember that polymicrobial and host-associated biofilms pose significant challenges in translating antibiofilm strategies into the clinical setting.

Many experimental studies, including those discussed in this review, focus on monospecies biofilm models; however, most clinically relevant biofilms are polymicrobial. They consist of multiple bacterial species or mixed communities of bacteria, fungi, and viruses, in which interspecies interactions promote increased antimicrobial tolerance and increased structural stability compared to monospecies biofilms [179,180]. Importantly, bacterial–fungal biofilms, such as those formed by *C. albicans* in association with Gram-(+) or Gram-(−) bacteria (e.g., in the oral cavity), are particularly resistant to antimicrobial treatment and are often associated with chronic wound and medical device-related infections [180,181]. The presence of biofilms in host-associated environments (e.g., chronic wounds, respiratory tract, and gastrointestinal tract), where microbial communities are strongly influenced by the host immunity, nutrient availability, and resident microbiota, is not without significance and causes such biofilms to exhibit phenotypes that differ remarkably from those observed in vitro, limiting the predictive value of simplified laboratory models [182,183].

BBR exhibits a broad spectrum of antimicrobial activity, which underlies its potential as an antibiotic adjuvant, but also raises consequential ecological concerns. In polymicrobial communities, this low-selectivity activity may not eliminate all microorganisms uniformly; instead, it may preferentially inhibit the growth of some species while allowing others to survive or expand. As a result, treatment may alter biofilm composition rather than completely eliminate it [180].

In host-associated environments, such as the gut or skin, these changes in microbiome composition can disrupt the resident microbiota and potentially contribute to dysbiosis. Currently, available information on the effects of BBR on the structure of microbial communities in vivo is limited and the available clinical data are insufficient to fully assess this potential risk [184,185]. Therefore, future studies should evaluate BBR-based combination therapies not only for antimicrobial efficacy but also for their impact on host-associated microbial ecosystems.

Therefore, future research should focus on several priority areas: 1. Further development and testing of nanoforms and local delivery systems (hydrogels, coatings, dressings) in infection models involving biofilm formation—particularly infections of orthopedic implants, vascular catheters, and chronic wounds. 2. Translational research combining BBR with antibiotics in clinically relevant settings—taking into consideration strain and virulence factor variability (e.g., EP expression), biofilm conditions, and its maturity. 3. Standardization of therapeutic doses depending on the delivery method—establishing minimum effective concentrations (with different therapeutic targets—prevention versus treatment). 4. A more detailed understanding of the pharmacokinetic properties and biodistribution of BBR, with emphasis on its potential toxicity (possible kernicterus and induction of premature uterine contractions) and interactions with cytochrome P450 substrates [178].

## 7. Conclusions

Biofilm-associated infections remain one of the most challenging problems in current medicine due to their natural tolerance to antimicrobial drugs, ability to avoid the host’s immune response, and strong association with MDR. The complex architecture of biofilm, combined with metabolic heterogeneity, horizontal gene transfer, and coordinated regulation via QS, significantly limits the effectiveness of conventional antibiotic therapies. As highlighted in this review, these characteristics not only contribute to treatment failures but also drive the persistence and recurrence of chronic and device-associated infections.

BBR appears to be a particularly promising natural compound with broad-spectrum antimicrobial activity and a unique, multidirectional mechanism of action. Unlike classic antibiotics, which often have a single target, BBR disrupts multiple bacterial processes, including cell wall and cell membrane integrity, nucleic acid and protein synthesis, EP activity, and cellular energy metabolism, while inducing oxidative stress in bacterial cells. This pleiotropic mechanism of action reduces the likelihood of rapid resistance development and positions BBR as a valuable candidate in the context of antimicrobial resistance. Current evidence strongly supports BBR’s role in disrupting the bacterial cell wall and membrane integrity, as evidenced by SEM/TEM imaging and comprehensive metabolomic profiles showing significant inhibition of peptidoglycan and teichoic acid synthesis. Similarly, its function as an EP inhibitor is well-validated through restored antibiotic sensitivity and observed binding within pump regions across various species. In contrast, the direct interaction of BBR with specific regulatory proteins like LasR, RhlR, or DNA polymerase III remains more speculative, as these mechanisms are primarily suggested by in silico docking studies and require further biochemical confirmation. Furthermore, the antibiofilm activity of BBR exhibits distinct patterns between Gram-(+) and Gram-(−) bacteria; while it primarily targets the cell wall architecture in Gram-(+), its efficacy in Gram-(−) is critically linked to its ability to bypass the outer membrane and inhibit RND-family EP.

This review highlights the significant potential of BBR in combating biofilm. By inhibiting early adhesion, disrupting QS-regulated pathways, impairing EPS production, and weakening mature biofilm structures, BBR effectively targets key stages of biofilm development. These properties are particularly relevant in the prevention and treatment of chronic, implant- and catheter-associated infections, where biofilm formation is a key determinant of clinical outcome. Literature data indicate that BBR can restore and even enhance the effectiveness of antibiotics against biofilm-forming MDR pathogens through synergistic interactions, EP inhibition, and modulation of the bacterial stress response. Such combination strategies may reduce the required antibiotic dose, limit adverse effects, and alleviate the selection pressure favoring resistant strains. Therefore, BBR-based combination therapies offer hope for the treatment of infections that are currently difficult or impossible to eradicate with antibiotics alone.

In addition, an important yet insufficiently explored aspect of the potential therapeutic application of BBR is its impact on the host microbiota. Owing to its broad-spectrum antibacterial activity and ability to modulate bacterial metabolism, BBR affects not only biofilm-forming pathogens but also the commensal microbiota, potentially leading to dysbiosis. However, due to its immunomodulatory and selective effects, BBR may modulate the host microbiota composition. Therefore, assessing the balance between therapeutic efficacy and the risk of dysbiosis induction should represent a key priority for future in vivo studies and clinical trials, particularly in the context of long-term and combination therapies.

Despite these encouraging findings, a number of challenges remain. Currently, the most robust data are derived from in vitro antibiofilm studies, which demonstrate significant molecular potential. Preclinical in vivo evidence, though promising in animal models, remains limited and insufficient to establish standardized clinical protocols. This clinical translation is further hindered by BBR’s inherently low systemic bioavailability, which results from its poor water solubility and extensive first-pass metabolism. Therefore, the clinical utility of BBR remains hypothetical and subject to future validation through controlled trials. At this stage of research, BBR should be viewed primarily as a lead compound or an adjuvant prototype rather than a ready-to-use therapeutic agent. Furthermore, the molecular mechanisms underlying the synergy between BBR and antibiotics and their specific effects on biofilm require further clarification, particularly in the context of multibacterial and host-associated biofilm. However, BBR is such a versatile and biologically credible adjunct in the fight against biofilm-associated and antibiotic-resistant infections that future research should prioritize standardized in vivo models, advanced drug delivery systems, and well-controlled clinical trials to fully exploit its therapeutic potential. The inclusion of BBR in multidimensional strategies to combat biofilm may significantly contribute to overcoming one of the most pressing challenges in modern antimicrobial therapy.

## Figures and Tables

**Table 1:** The summary of biofilm formation stages with regard to current virulence/resistance factors and its implications to mentioned processes.

Attachment of Bacterial Cells	
MSCRAMMs (e.g., protein A, fibronectin binding proteins, ClfA/B, M protein, FbsA/B)	adhesion of Gram-(+) bacteria to host and environmental ECM components
Fimbriae and adhesins (FimA, FimH, curli, YadA, OprF), pili (PilA)	adhesion of Gram-(−) bacteria to ECM structures
**Microcolony formation and maturation of biofilm**	
EPS polysaccharides (PIA-*icaADBC*, PEL, alginate)	intercellular adhesion; reduced antibiotics penetration; increased stress tolerance
Other EPS components (eDNA, proteins, lipids, Saccharides)	biofilm stabilization; selective permeability; immune evasion; metabolite and waste transport
QS and regulatory systems	regulation of virulence and biofilm-associated gene expression
**Dispersal of biofilm**	
Specific proteases, DNases, saccharidases	enzymatic degradation of ECM
QS modulation	induction of dispersal mechanisms (e.g., rhamnolipids synthesis, PEL inhibition, activation of lytic enzymes)

Abbreviations: ClfA/B—clumping factor A/B; ECM—extracellular matrix; eDNA—extracellular DNA; EPS—extracellular polymeric substances; FbsA/B—fibrinogen binding proteins A/B; FimA/H—fimbriae type A/H; MSCRAMM—microbial surface components recognizing adhesive matrix molecules; OprF—specific outer membrane protein present in *Pseudomonas aeruginosa*; PEL—component of *P. aeruginosa* EPS; PIA—polysaccharide intercellular adhesin; QS—quorum sensing; YadA—*Yersinia* adhesin A.

**Table 2:** Biofilm-related virulence and resistance mechanisms in clinical infections.

Biofilm-Related Virulence/Resistance	Type of Infection and Its Mechanism	References
**EPS: proteins, polysaccharides, eDNA**	Vascular catheter-associated infections (Gram-(+)/(−) etiology)-reduced antibiotic penetration and sequestration via cationic interactions within the matrix	[75,76,77,78,79,80,109]
**Persistent cells populations**	Chronic pulmonary colonization and recurrent *P. aeruginosa* infections in cystic fibrosis–phenotypic tolerance to antibiotic targeting actively dividing cells	[110]
**Horizontal genes transfer via MGEs**	Urinary catheter-associated infections of non-fermenters and *Enterobacterales* etiology-enhanced retention and dissemination of MDR plasmids in biofilm vs. planktonic populations	[94,95]
**Local antibiotic activation**	Chronic wound infections with mixed-species biofilm-accumulation of β-lactamases in the matrix and cross-resistance mechanisms (e.g., EP, *vanA* transfer)	[79,93,95,96,97,98]

Abbreviations: eDNA—extracellular DNA; EP—efflux pump; EPS—extracellular polymeric substances; MDR-multidrug-resistant; MGEs—mobile genome elements; *vanA*—gene associated with resistance to glycopeptides, e.g., vancomycin.

**Table 3:** Key biofilm-regulatory genes in *S. aureus* and their roles relevant to BBR-mediated inhibition of biofilm formation and dispersal.

Gene	Role in Biofilm Formation	Biofilm Stage Affected
*cidA*	influences cell lysis and release of cytoplasmic contents; enables eDNA release –> early biofilm formation [128]	Initial adhesion and early biofilm formation
*icaA*	synthesizing polysaccharide intercellular adhesin (PIA) –> intercellular adhesion, biofilm accumulation, and formation of the thick multilayer matrix [129]	Biofilm maturation and matrix accumulation
*sarA*	QS regulatory system –> biofilm dispersal (when downregulated) or stabilization of biofilm (when upregulated) [130]	Maturation and dispersal phases
*clfA*	encoding a cell-wall-anchored adhesin that binds fibrinogen –> initial adhesion [131]	Initial adhesion
*hla*	encoding alpha-hemolysin, a pore-forming cytotoxin –> biofilm dispersal by damaging host barriers [132]	Biofilm dispersal
*agr*	QS regulatory system –> stabilization of biofilm (when downregulated) or biofilm dispersal (when upregulated) [133]	Maturation and dispersal phases

Abbreviations: eDNA—extracellular DNA; QS—quorum sensing.

**Table 4:** Types of adjuvants and their role in synergistic interaction with antibiotics.

Type of Adjuvant	Mechanism of Action	Synergistic Effects with Antibiotics	Source
**Direct adjuvants**	Inhibition of bacterial resistance mechanisms Blocking antibiotic-degrading enzymes (e.g., β-lactamases) Inhibition of EP	Reduction of antibiotic MIC value Enhanced activity against MDR strains	[102,152,153,154]
**Indirect adjuvants**	Modification of physicochemical conditions Increased bacterial membrane permeability Disruption of biofilm structure	Improved antibiotic penetration More effective eradication of biofilm-associated bacteria	[155]
**Host-modulating adjuvants**	Enhancement of the host immune response Increased phagocytic activity Induction of cytokine and chemokine production	Indirect support of pathogen elimination Improved immune-mediated bacterial clearance	[102,155]

Abbreviations: EP—efflux pump; MIC—minimal inhibitory concentration; MDR—multidrug-resistant.

**Table 5:** Combinations of BBR with antibiotics leading to increased therapeutic effect and its impact on biofilm.

Pathogen	BBR + Antibiotic	Synergy Mechanism	Reference
MRSA	BBR + ciprofloxacin/tobramycin	EP inhibition	[50]
*P. aeruginosa*	BBR + imipenem BBR + azithromycin	EP inhibition Reduced alginate production and biofilm matrix modulation	[140,157]
Biofilm-forming GN bacteria (e.g., *E. coli*, *K. pneumoniae*, *Salmonella* spp.	BBR + β-lactams/Quinolones/Aminoglycosides/Tetracyclines/Macrolides/Lincosamides/Fusidic acid	Biofilm disruption—inhibition of adhesion, microcolony formation and maturation	[50]
every biofilm-forming strain	BBR + any antibiotic	host-mediated effect: suppression of proinflammatory cytokines, enhancement of immune response	[158,159]

Abbreviations: BBR—berberine; EP—efflux pump.

## Data Availability

No new data were created or analyzed in this study. Data sharing is nor applicable to this article. The original contributions presented in this study are included in the article. Further inquiries can be directed to the corresponding author.
